# One-Step Fabrication of 2.5D CuMoO_x_ Interdigital Microelectrodes Using Numerically Controlled Electric Discharge Machining for Coplanar Micro-Supercapacitors

**DOI:** 10.3390/mi15111319

**Published:** 2024-10-29

**Authors:** Shunqi Yang, Ri Chen, Fu Huang, Wenxia Wang, Igor Zhitomirsky

**Affiliations:** 1 College of Informatics, Huazhong Agricultural University, Wuhan 430070, China; yyssqqq@webmail.hzau.edu.cn; 2 Department of Mechatronic Engineering, Guangdong Polytechnic Normal University, Guangzhou 510665, China; 3 Department of Biomedical and Pharmaceutical Sciences, Guangdong University of Technology, Guangzhou 510006, China; fewwxia@gdut.edu.cn; 4 School of Materials Science and Engineering, McMaster University, Hamilton, ON L8S 4L7, Canada; zhitom@mcmaster.ca

**Keywords:** coplanar micro-supercapacitors, numerically controlled electric discharging machining, binary metal oxides, machining voltage, CuMoO_x_

## Abstract

With the increasing market demands for wearable and portable electronic devices, binary metal oxides (BMOs) with a remarkable capacity and good structure stability have been considered as a promising candidate for fabricating coplanar micro-supercapacitors (CMSCs), serving as the power source. However, the current fabrication methods for BMO microelectrodes are complex, which greatly hinder their further development and application in BMO CMSCs. Herein, the one-step fabrication of 2.5D CuMoO_x_-based CMSCs (CuMoCMSCs) has been realized by numerically controlled electric discharge machining (NCEDM) for the first time. In addition, the controllable capacity of CuMoCMSCs has been achieved by adjusting the NCEDM-machining voltage. The CuMoCMSCs machined by a machining voltage of 60 V (CuMoCMSCs60) showed the best performance. The fabricated CuMoCMSCs60 with binary metal oxides could operate at an ultra-high scanning rate of 10 V s^−1^, and gained a capacity of 40.3 mF cm^−2^ (1.1 mA cm^−2^), which is more than 4 times higher than that of MoO_x_-based CMSCs (MoCMSCs60) with a single metal oxide. This is because CuMoO_x_ BMOs materials overcome the poor electroconductivity problem of the MoO_x_ single metal oxide. This one-step and numerically controlled fabrication technique developed in this research opens a new vision for preparing BMO materials, BMO microelectrodes, and BMO microdevices in an environmental, automatic, and intelligent way.

## 1. Introduction

In recent years, there is an increasing market demand for wearable, portable, and implantable electronic devices in our daily life for various fields, such as micro-robots, micro-sensors, micro-actuators, and micro-electromechanical systems [1,2,3,4,5]. As these intelligent electronics are in a miniaturized size, it is necessary to fabricate microscale energy storage devices which could be efficiently integrated with other small electronic devices to meet the vision of the Internet of Things (IoT) [6,7,8,9]. Unlike other microscale energy storage devices of nano-generators and solar-cells which are periodic, intermittent, and unstable, micro-batteries and micro-supercapacitors have been widely used as the power supply for various applications. In contrast to micro-supercapacitors, micro-batteries suffer from the problems of their low safety, unstable cyclic stability, and unsatisfied power density [2,10,11,12]. Therefore, micro-supercapacitors (MSCs) with the merits of high safety, stable cyclic stability, and satisfied power density have been broadly adopted as the power units for microelectronics application [13,14]. Unfortunately, conventional sandwich-shape micro-supercapacitors assembled with two electrodes separated by a separator inevitably result in large devices size, which are difficult for the integration with other electronics, hindering their broad application in power units [11,15,16]. In contrast, representative coplanar micro-supercapacitors (CMSCs) with microelectrodes arrays constructed in the same plane without using an extra separator demonstrate their promising potential and great compatibility for the integrated manufacturing with other microelectronics [17,18,19].

The electrochemical performance of CMSCs is greatly dependent on their electrode materials, electrolyte, and device architecture. Among these three main parameters, electrode material selection is the most important one for fabricating efficient CMSCs. Currently, carbon-related materials (like graphene [20,21], the carbon nanotube [22,23], and porous carbon [24,25]), single metal oxides/hydroxides (such as MnO_2_ [26], VO_0_._2_ [27], RuO_2_ [28,29], MoO_x_ [6], Co_3_O_4_ [30,31], CuO [32,33], Fe_1−x_O [7], and FeOOH [34]), and conducting polymers (like poly(3,4-ethylenedioxythiophene) [35,36], polypyrrole [37,38], and polyaniline [39]) are the most popular in the supercapacitors application. However, carbon-based materials show a great limitation in their low energy density whereas conducting polymers are hindered by their low structural stability. Therefore, single metal oxides/hydroxides providing high capacitance in theory and abundant materials sources attract intense attention from researchers [40,41]. Unfortunately, the poor electroconductivity of single metal oxides/hydroxides limits their further development in various fields. In recent years, more researchers’ interest has been focused on developing single metal oxides/hydroxides with element doping, nanostructures, and vacancies, preparing single metal oxide/hydroxide-based composites using high-conducting additives, and synthesizing binary metal oxides (BMOs) combined with two different single metal oxide [42,43,44]. Among these strategies, BMOs show great potential for constructing supercapacitor electrodes with excellent capacitive characteristics as they could provide multiple oxidation states for promoting the transport efficiency of a redox charge [45]. For instance, Mohammadi et al. used a hydrothermal strategy to prepare flexible supercapacitor electrodes with dandelion-shape CoMoO_4_–CoMoO_4_ core-shell nanostructure materials, providing a superior capacity (1548 F g^−1^ at 1 A g^−1^) with an excellent capacitance retention (94% for 5000 cycles). The core-shell nanostructure and multiple oxidation state of CoMoO_4_–CoMoO_4_ provided plentiful active sites as well as fastened the electronic transport speed, which lead to a good capacitive behavior of the whole electrodes. Xu et al. [46] combined a microwave-aided hydrothermal method and calcination to prepare ZnMoO_4_ nanosheet arrays on nickel foam with 3D frameworks for a supercapacitor electrode, offering a good capacity value of 1212 F g^−1^ at 1 A g^−1^ and a superior rate performance. The 3D frameworks, nanosheet structure, and polyvalent state of ZnMoO_4_ facilitated reduced electronic–ionic migration paths and, thus, enhanced the electrodes performance. Feng et al. [47] utilized a combined method of chemical vapor deposition and hydrothermal reaction to prepared NiMoO_4_–graphene composite electrodes, which provided a satisfied capacitance value (1913 F g^−1^ at 1 A g^−1^). The introduction of high-electroconductivity filler graphene and multiple oxidation states greatly boosted the electroconductivity of NiMoO_4,_ and, thus, an enhanced performance was gained. Sivakumar et al. [48] synthesized oxygen-defect-enriched flower-like NiMoO_4_ (O_d_-NiMoO_4_) nanomaterials for the hybrid supercapacitor using a combination of the hydrothermal method and thermal treatment strategy in a specific argon-gas environment. It was proven that Od-NiMoO_4_-based electrodes offered a higher capacity (789 mAh g^−1^ at 1 A g^−1^) and better rate performance with respect to that of NiMoO_4_ with oxygen vacancy. The is benefiting from the enhanced electroconductivity of NiMoO_4_ triggered by oxygen defects, and a high surface area, as well as multiple oxidation states. Recently, copper molybdates receive concentrated attention because of its high theoretical capacitance, good morphologies, eco-friendliness, and relatively low cost [49]. For instance, Shenjini et al. grew CuMoO_4_ on N-doped reduced graphene oxide (NrGO) to form a CuMoO_4_–NrGO nanocomposite for supercapacitor application using a combined technique of the modified Hummer’s method, calcined treatment, and hydrothermal strategy. The results suggested that good redox behavior was achieved by the CuMoO_4_–NrGO nanocomposite because high-electroconductivity NrGO and the poly-oxidation state of CuMoO_4_ improved the electroconductivity of the CuMoO_4_–NrGO electrodes. Alagarsamy et al. [50] also combined the hydrothermal method and ultrasonic methods to synthesize CuMoO_4_ on graphene-oxide nanoribbons (GON) for obtaining CuMoO_4_–GON composite electrodes, which offered a high capacitance value (865 F g^−1^ at 1 A g^−1^). The benefits from the high surface area, multivalent state, and conductive-additive assistance of the CuMoO_4_–GON composite, which provided sufficient active sites and conductive paths for electron/ion movement. Although a great amount of effort has been tried to boost the electrochemical performance of copper molybdates, their complex fabrication procedures greatly hinder their development and application. Particularly, there is a lack of reports on the one-step fabrication of copper-molybdate-based integrated microelectrodes for CMSC application. Moreover, the size and shape of the microelectrodes also play an important role in the capacitive performance of CMSCs. It was reported that CMSCs with an interdigital shape facilitated transporting electrolyte ions in a transverse direction, which boosted the ion transfer efficiency. In addition, the interdigital CMSCs are easy to be integrated with other electronics on the plane. As a result, interdigital CMSCs attracted concentrated attention from researchers [11,51]. Therefore, this encourages us to concentrate our research interest on simplifying the manufacturing process of CuMoO_x_-binary-metal-oxide-based integrated microelectrodes with a remarkable redox behavior for CMSCs with an interdigital shape.

In this research, the one-step fabrication of 2.5D CuMoO_x_-binary-metal-oxide-based interdigital CMSCs (CuMoCMSCs) with a customizable geometric shape has been realized by machining the Mo metal substrate using copper wire via numerically controlled electric discharge machining (NCEDM) for the first time. For comparison, the one-step fabrication of 3D MoO_x_-single-metal-oxide-based interdigital CMSCs (MoCMSCs) has also been fabricated by machining the Mo metal substrate using Mo wire via NCEDM. The proposed NCEDM technique is versatile for designing and fabricating 2.5D binder-free integrated electrodes composed of a 2.5D metal current collector and metal-oxide-based active materials. Moreover, the NCEDM technique facilitates tailoring the surface morphologies via controlling the machining voltage. It could also control the composition of the metal-oxide-based active materials by the material selection of the machining tool. The investigated results demonstrated that the fabricated CuMoCMSCs60 with binary metal oxides could operate at an ultra-high scanning rate of 10 V s^−1^, and gained a capacity of 40.3 mF cm^−2^ (1.1 mA cm^−2^), which is more than 4 times higher than that of MoCMSCs60 with single metal oxides. This is because CuMoO_x_ with binary metal oxides overcome the poor electroconductivity problem of MoO_x_ with a single metal oxide. The most important thing is that the CMSCs are machined by one-step NCEDM, which is free of binders, conducting fillers, noble materials, extra current collectors, and toxic solutions. This one-step and numerically controlled fabrication technique developed in this research opens a new vision for preparing BMO materials, BMO microelectrodes, and BMO microdevices in an environmental, automatic, and intelligent way.

## 2. Details of Experiments

### 2.1. Materials and Methods

Molybdenum substrates for NCEDM were purchased from the Metal-materials Corporation of Qing He Li Sheng, Xingtai, China. The molybdenum wires and copper wire used as machining tool for NCEDM were achieved by the Guang Ming Company Corporation of Jin Dui Cheng, Zibo, China and 1 M KOH solutions used for electrochemical characterization of CMSCs were bought from Kell-chemical Technology Corporation, Guangzhou, China.

### 2.2. Preparation of Metal-Oxide-Based Microelectrodes for CMSCs

All the machining processes of NCEDM were performed with a wire electric discharge machining machine called HB 400C, which was purchased from the company of Suzhou Sanguang Technology, Suzhou New District, China. Firstly, one-step fabrication of 2.5D CuMoCMSCs with interdigital shape has been realized by machining Mo metal substrate using copper wire as machining tool via NCEDM. The CuMoCMSCs machined by machining voltages of 60, 80, and 100 V were marked as CuMoCMSCs60, CuMoCMSCs80, and CuMoCMSCs100, respectively. For comparison, one-step fabrication of 2.5D MoCMSCs with interdigital structure machined by 60 V (labeled as MoCMSCs60) has been fabricated by machining Mo metal substrate using Mo wire as the machining tool via NCEDM. Other NCEDM machining parameters for fabricating MoCMSCs60, CuMoCMSCs60, CuMoCMSCs80, and CuMoCMSCs100 devices were set as follows: processing current (2 A), time on of the discharge pulse (24 μs), duty cycle (4:1), and dielectric fluid (deionized water). The diameter of Mo wire is 180 μm, whereas the diameter of Mo wire is 200 μm.

### 2.3. Material Characterization for CMSC Microelectrodes and Microdevices

The samples’ surface morphology and structure were investigated using scanning electron microscope (SEM, TESCAN, Brno, Czech Republic) with an analyzer of energy-dispersive spectroscopy (EDS, TESCAN MIRA LMS, Brno, Czech Republic), X-ray photoelectron spectroscopy (XPS, Thermo Scientific K-Alpha, Waltham, MA, USA), and X-ray diffraction (XRD, Rigaku Smartlab, Tokyo, Japan). The XRD testing was performed by Cu Kα radiation with a scanning rate of 2° min^−1^. Cyclic voltammetry (CV) and Galvanostatic charge/discharge (GCD) characterization of MoCMSCs60, CuMoCMSCs60, CuMoCMSCs80, and CuMoCMSCs100 was evaluated using a CHI660E potentiostat (Chenhua, Shanghai, China), whereas the characterized area for all the devices was controlled at 1 cm^−2^. The voltage windows of MoCMSCs60, CuMoCMSCs60, CuMoCMSCs80, and CuMoCMSCs100 were set at 0–0.6 V, and their CV and GCD studies were performed at 5–10, 000 mV s^−1^, and 1.1–5 mA cm^−2^, respectively.

## 3. Results and Discussion

**Figure 1:**
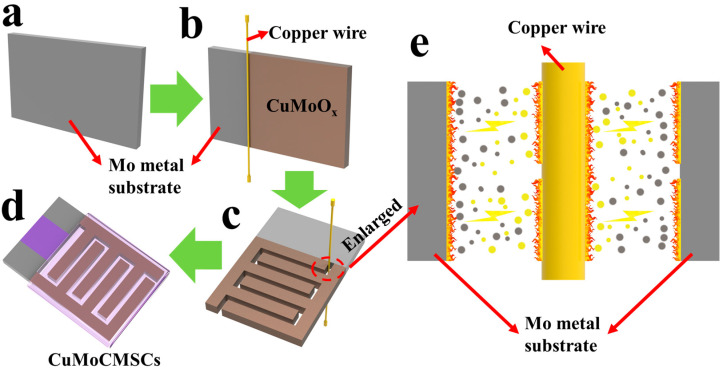
(**a**) Mo metal substrate before NCEDM, (**b**) surface of Mo metal substrate machined by NCEDM to achieve CuMoO_x_ binary metal oxides based integrated electrodes, (**c**) the machining electrodes machined into interdigital shape, (**d**) interdigital CuMoCMSCs, and (**e**) the enlarged schematic of discharge channel during NCEDM processing.

Figure 1 makes a description about the fabricated processing of 2.5D binder-free interdigital-integrated CuMoCMSCs composed of a Mo current collector and CuMoO_x_ binary metal oxides active materials, which are achieved simultaneously using copper wires as the machining tool for machining the Mo current collector through the NCEDM technique. It could be easily found that the Mo metal substrate serves as the current collector and provides a Mo material source for synthesizing CuMoO_x_ binary metal oxides, whereas the copper wire plays the part of machining tools and offers Cu material sources for the synthesis of CuMoO_x_ binary metal oxides. When the applied voltage between the copper wire and Mo substrate is strong enough, the spark in this narrow gap would be triggered and generates a larger amount of heat resulting in the evaporation and melting of copper wire and Mo metal substrates. As the applied machining voltage is periodic, when it is off and the spark would be shut down, the melting copper and molybdenum would be rapidly cooled down by the machining fluid.

**Figure 2:**
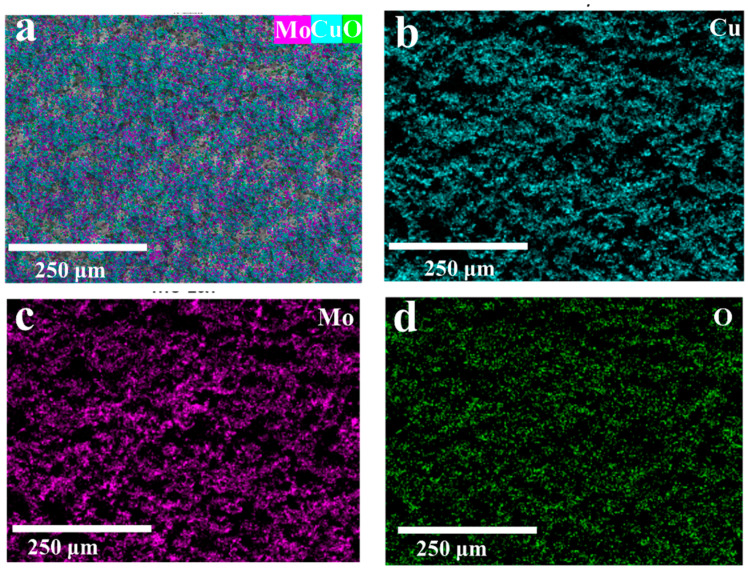
SEM-EDS analysis results for CuMoO_x_: distribution diagram of different elements—(**a**) Cu, Mo, and O; (**b**) Cu; (**c**) Mo; and (**d**) O.

Meanwhile, CuMoO_x_ binary metal oxide active materials would be formed on the surface of the molybdenum metal substrate to achieve binder-free CuMoO_x_ integrated electrodes, which is good for energy storage. Benefiting from the computer-aided design of NCEDM, these CuMoO_x_-integrated electrodes would be subsequently patterned into an interdigital shape to obtain 2.5D binder-free interdigital-integrated CuMoCMSCs. SEM equipped with an EDS analyzer was utilized to study the distribution diagram of elements for CuMoO_x_-integrated electrodes achieved by NCEDM (Figure 2). The examined results illustrate that the elements of Cu, Mo, and O were homogeneously distributed on the CuMoO_x_-integrated electrodes surface, which proved that NCEDM facilitates the one-step synthesis of CuMoO_x_ binary metal oxides on the Mo current collector with good quality. Moreover, this integrated electrode design is free of the binder, which is helpful for fastening the electronic movement efficiency.

**Figure 3:**
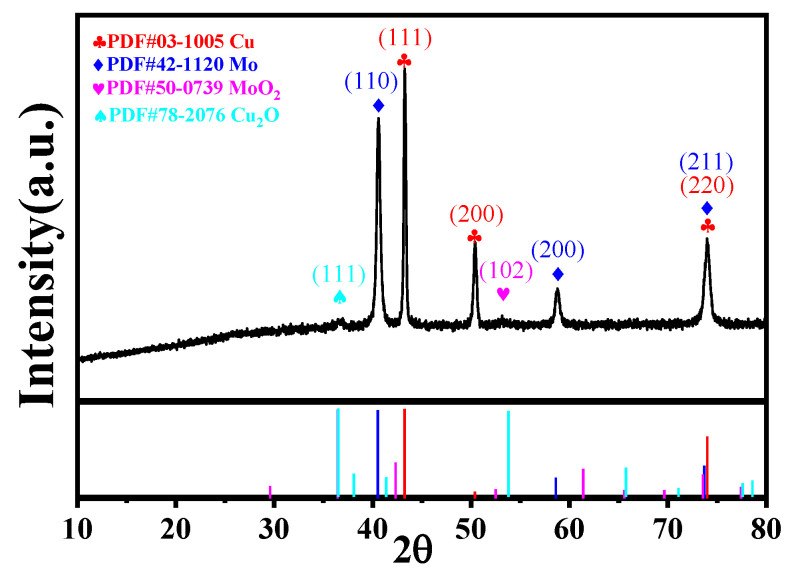
XRD patterns equipped with PDF card number for CuMoO_x_ prepared by one-step NCEDM.

**Figure 4:**
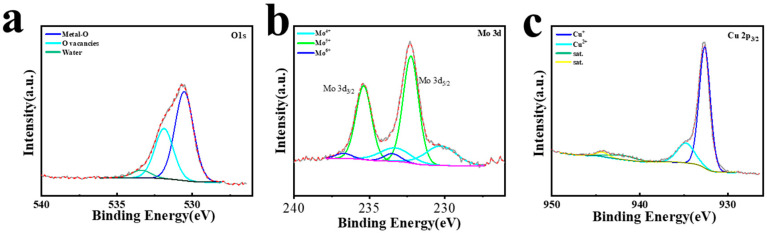
XPS patterns of (**a**) O1s, (**b**) Mo 3d, and (**c**) Cu 2p_3/2_ for CuMoO_x_ prepared by one-step NCEDM.

**Figure 5:**
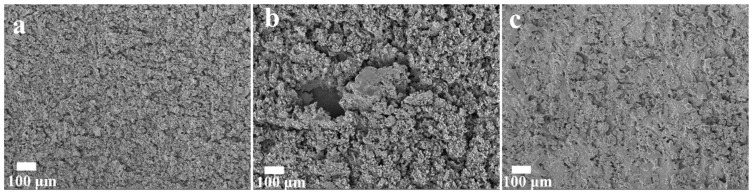
SEM pictures of electrode materials for (**a**) CuMoCMSCs60, (**b**) CuMoCMSCs80, and (**c**) CuMoCMSCs100 fabricated by one-step NCEDM.

Figure 3 shows the XRD diffraction pattern of CuMoO_x_. The points peak at 43.3°, 50.4°, and 74.1°, representing the planes of (111), (200), and (220) for Cu (PDF#03-1005), respectively [52]. The peaks at 40.6°, 58.6°, and 73.9° correspond to the (110), (200), and (211) planes of Mo (PDF#42-1120) [53]. Additionally, weak peaks observed at 36.7° and 53.1° could be attributed to the (111) plane of Cu_2_O (PDF#78-2076) and the 102 plane of MoO_2_ (PDF#50-0739), respectively [54,55]. Figure 4a illustrates that the O 1s curve could be deconvoluted into three distinct oxygen species: metal–oxygen bonds of 530.5 eV, oxygen vacancies of 531.9 eV, and adsorbed water on the surface located at 533.4 eV [56]. Figure 4b displays the Mo 3d spectrum, with peaks at 230.3 eV and 233.3 eV assigned to Mo 3d_5/2_ and Mo 3d_3/2_ of Mo^4+^, respectively. Peaks at 232.3 eV and 235.4 eV corresponding to Mo 3d_5/2_ and Mo 3d_3/2_ belonged to Mo^5+^, while those at 233.5 eV and 236.7 eV stand for Mo 3d_5/2_ and Mo 3d_3/2_ of Mo^6+^ [57]. Figure 4c presents the Cu 2p_3/2_ XPS curve, revealing peaks at 932.7 eV and 934.8 eV, with respect to Cu^+^ and Cu^2+^, respectively, while the weak peaks observed near 943 eV and 946 eV correspond to satellite peaks [58,59]. This CuMoO_x_ active material equipped with Mo^4+^, Mo^5+^, Mo^6+^, Cu^+^, and Cu^2+^ facilitates the fast electronic transfer rate, which is beneficial for boosting redox reaction kinetics and, thus, enhancing the microdevice performance. SEM studies were carried out to investigate the influence of machining voltages operated by NCEDM morphologies of CuMoO_x_ binary metal oxides. Figure 5a proved that the particles of CuMoO_x_ prepared at a low voltage of 60 V displayed a uniform distribution on the Mo current collector and contain enriched micro-/nanostructures, which are in favor of ion accessibility for charge storage. As the machining voltage increases to 80 V, the CuMoO_x_ particles showed a trend of agglomerations and sparse distribution (Figure 5b). When the machining voltage increases to 100 V, the largest amount of heat was generated in the spark channel and resulted in the severe agglomeration of CuMoO_x_ particles, which hindered the ion access on the particles’ surface (Figure 5c).

In order to further demonstrate the merit of CuMoO_x_ binary metal oxides, the MoO_x_ single metal oxide was also fabricated by the NCEDM strategy using Mo wire as the machining tool. Figure S1a showed the XRD profile for the MoO_x_ single metal oxide, which proved the coexistence of Mo, MoO_2_, and MoO_3_ in the Mo–MoO_x_ integrated electrodes. High-intensity diffraction peaks at 73.68°, 58.6°, and 40.5° are in good agreement with the plane of (211), (200), and (110), belonging to Mo (JCPDS card 42-1120) [60]. The peak appearing at 25.96° stands for the plane of (200) belonging to MoO_3_ (JCPDS card 47-1081) [61]. Furthermore, the corresponding peaks located at 36.7° and 53.5° were indexed to the planes of (100) and (102) for MoO_2_ (JCPDS card 50-0739), respectively [62]. Figure S1b,c illustrated the XPS results of the Mo–MoO_x_ electrode produced by the NCEDM method. Figure S1b showed that the peaks observed at 232.83 eV and 229.48 eV, respectively, stand for Mo 3d_3/2_ and Mo 3d_5/2_, which are the characteristic peaks of Mo^4+^. The peaks located at 236.06 eV and 232.88 eV are, respectively, corresponding to Mo 3d_3/2_ and Mo 3d_5/2_ indexed to Mo^6+^, providing the evidence for the synthesis of MoO_3_. In addition, the binding energies of 233.90 eV and 230.97 eV represent Mo 3d_3/2_ and Mo 3d_5/2_ of Mo^5+^, respectively, resulting from the reduction in MoO_3_ [63,64,65,66]. Figure S1c presents the O1s profile, which could be deconvoluted into three distinct peaks located at 530.2, 531.6, and 532.9 eV. These peaks correspond to the metal–O bond, oxygen vacancies, and surface-adsorbed water, respectively [67]. In addition, the SEM results (Figure S2) proved that NCEDM facilitated the fabricating of MoO_x_-based electrodes with a rough surface covered by lots of small particles, which provided sufficient active sites for energy storage. The results further demonstrated the NCEDM technique could facilitate the fabrication of various metal-oxide-based electrodes for broad applications.

**Figure 6:**
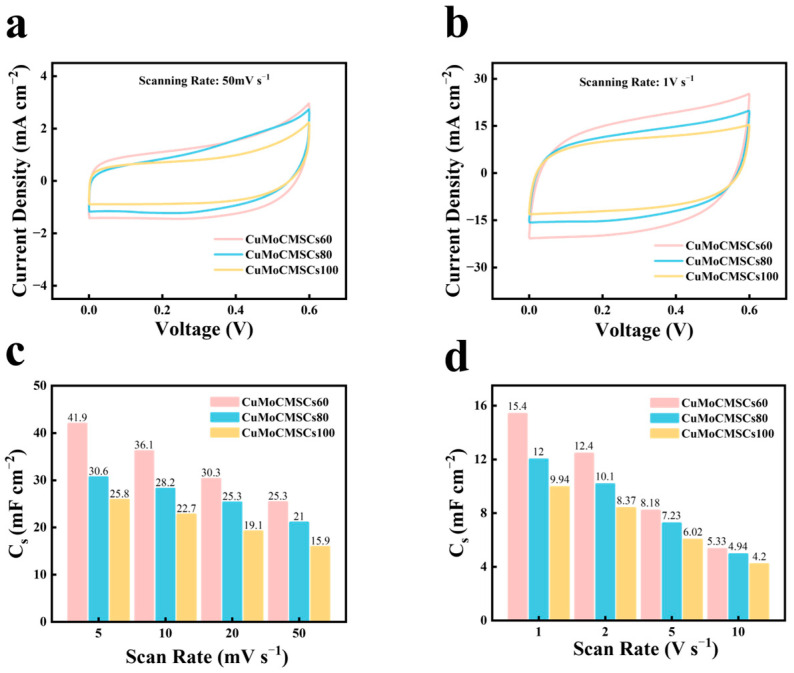
CVs of CuMoCMSCs60, CuMoCMSCs80, and CuMoCMSCs100 at (**a**) 50 mV s^−1^ and (**b**) 1 V s^−1^, respectively; capacitance in comparison for CuMoCMSCs60, CuMoCMSCs80, and CuMoCMSCs100 within (**c**) 5–50 mV s^−1^ and (**d**) 1–10 V s^−1^, respectively.

The NCEDM strategy could not only fabricate MSCs with different shape but also tailor the surface morphology of CuMoO_x_ active materials by adjusting the machining voltage, and thus manufacture CuMoMSCs with a controlled electrochemical performance. The CV testing results for CuMoCMSCs60, CuMoCMSCs80, and CuMoCMSCs100 manufactured with a corresponding machining voltage of 60, 80, and 100 V are displayed in Figure 6. It is shown that CuMoCMSCs60 showed the largest current density, whereas CuMoCMSCs100 obtained smallest current density both at 50 mV s^−1^ (Figure 6a) and 1 V s^−1^ (Figure 6b). All the three microdevices obtained a rectangle-shaped CV profiles, which verifies that the CuMoO_x_-based CMSCs prepared by NCEDM showed a good capacitive performance. Figure 6c,d demonstrates that CuMoCMSCs60 achieved the largest capacitance, whereas CuMoCMSCs100 obtained the smallest value. GCD testing was further performed to verify the capacitive performance of CuMoCMSCs60, CuMoCMSCs80, and CuMoCMSCs100.

**Figure 7:**
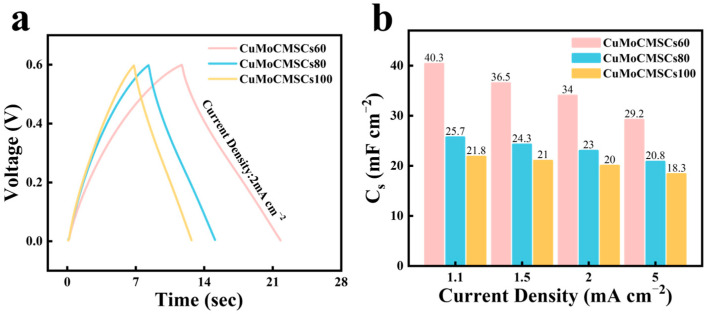
(**a**) GCD profiles in comparison for CuMoCMSCs60, CuMoCMSCs80, and CuMoCMSCs100 at 2 mA cm^−2^; and (**b**) capacitance for CuMoCMSCs60, CuMoCMSCs80, and CuMoCMSCs100.

Figure 7a proved that all these three devices achieved symmetric GCD profiles, which indicated good capacitive behavior was gained. Moreover, CuMoCMSCs60 showed the longest discharging time, whereas CuMoCMSCs100 displayed the shortest discharging time at 2 mA cm^−2^, which means CuMoCMSCs60 achieved the best capacitive performance. This is because the particles of CuMoO_x_ prepared at a low voltage of 60 V displayed a uniform distribution on the Mo current collector and contain enriched micro-/nanostructures, which are in favor of ion accessibility for charge storage (presented in Figure 5a). When the machining voltage increases from 60 to 100 V, a relatively larger amount of heat was generated in the spark channel and resulted in a more severe particle agglomeration of CuMoO_x_ (Figure 5b,c), which hindered ion access on the particles’ surface. Moreover, compared to CuMoCMSCs80 and CuMoCMSCs100, CuMoCMSCs60 gained a superior rate performance of 72.5% (from 1.1 to 5 mA cm^−2^) and a high capacitance of 40.3 mF cm^−2^ at 1.1 mA cm^−2^ (Figure 7b).

**Figure 8:**
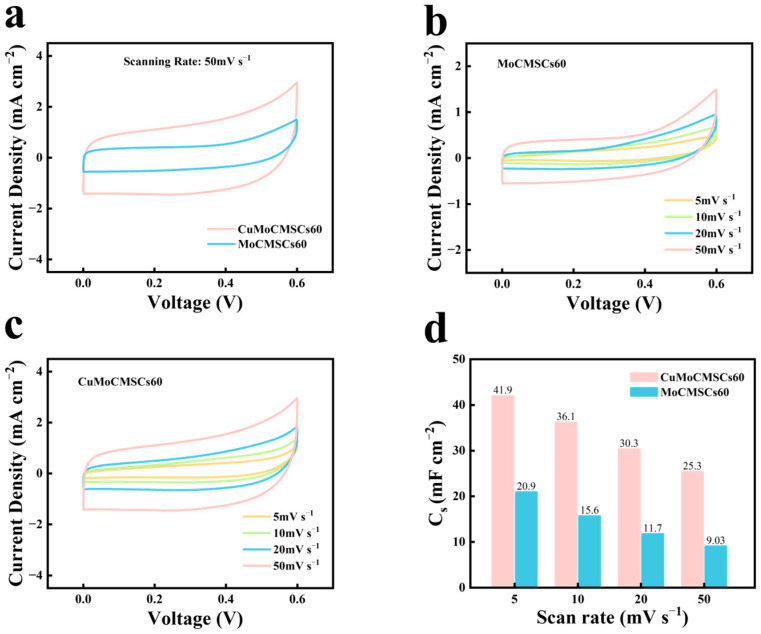
CVs of (**a**) MoCMSCs60 and CuMoCMSCs60 at 50 mV s^−1^, (**b**) MoCMSCs60 (5–50 mV s^−1^) and (**c**) CuMoCMSCs60 (5–50 mV s^−1^), and (**d**) capacitance in comparison for MoCMSCs60 and CuMoCMSCs60.

Figure 8 summarized the CV investigations of MoCMSCs60 and CuMoCMSCs60 machined by the NCEDM technique at a low potential range of 5–50 mV s^−1^. Figure 8a demonstrates that the current density of CuMoCMSCs60 is much larger than that of MoCMSCs60 at 50 mV s^−1^, which means CuMoCMSCs60 gained a better capacitive performance. Figure 8a,b depicts that both the devices of MoCMSCs60 and CuMoCMSCs60 showed a rectangle-shaped CV curve, which indicates that both of them obtained a good capacitive performance, which is stemming from the 2.5D binder-free electrode architecture design, the synthesis of micro-/nanostructure particles, and the introduction of oxygen vacancy. It could be observed that CuMoCMSCs60 obtained a high capacitance of 41.9 mF cm^−2^ at 5 mV s^−1^, which is around two times larger than that of MoCMSCs60. In addition, CuMoCMSCs60 gained a higher capacitance than those supercapacitors presented in the literature, such as the NiWO_4_//AC (activated carbon) asymmetric flexible supercapacitor prepared by the wet chemical route and slurry impregnation (17.01 mF cm^−2^) [68], V_2_O_5_-rGO (reduced graphene oxide) solid-state flexible MSCs fabricated by spray coating and sputtering (24 mF cm^−2^) [69], MnO_2_//NiCo_2_O_4_ MSCs prepared by electrodeposition, electron beam evaporation, and spin-coating (5.36 mF cm^−2^) [70], MXene MSCs manufactured by laser machining and spray coating (23 mF cm^−2^) [71], CuCo_2_O_4_–CNT(carbon nanotube) MSCs prepared by screen printing and the hydrothermal method (10.88 mF cm^−2^) [72], activated carbon MSCs manufactured by inkjet printing, photolithography, and chemical vapor deposition (5.1 mF cm^−2^) [73], MnO_2_-carbon MSCs prepare by screen printing and the hydrothermal strategy (7.04 mF cm^−2^) [74], graphene MSCs fabricated by spin coating and lithography (0.08 mF cm^−2^) [20], HfO_2_-graphene MSCs prepared by laser engraving and sputtered deposition (6.4 mF cm^−2^) [75], and rGO MSCs manufactured via laser radiation and vacuum filtration (0.51 mF cm^−2^) [76].

**Figure 9:**
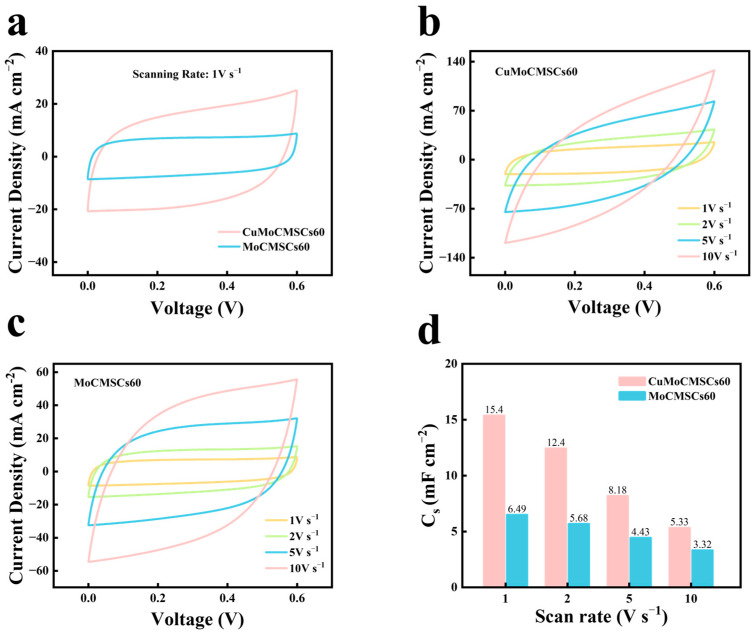
CVs of (**a**) MoCMSCs60 and CuMoCMSCs60 at 1 V s^−1^, (**b**) MoCMSCs60 (1–10 V s^−1^) and (**c**) CuMoCMSCs60 (1–10 V s^−1^), and (**d**) capacitance in comparison for MoCMSCs60 and CuMoCMSCs60.

Figure 9 demonstrates the CV investigations of MoCMSCs60 and CuMoCMSCs60 machined by the NCEDM strategy at a super-high potential range of 1–10 V s^−1^. Figure 9a depicts that the current density of CuMoCMSCs60 is much larger than that of MoCMSCs60 at 1 V s^−1^, which is consistent with the results presented in Figure 8a.

**Figure 10:**
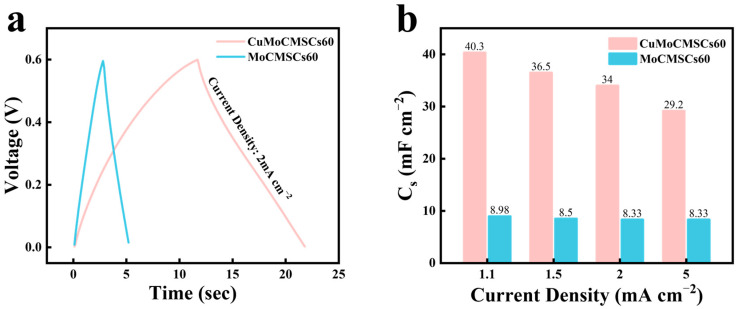
(**a**) GCD profiles in comparison for MoCMSCs60 and CuMoCMSCs60 at 2 mA cm^−2^; and (**b**) capacitance in comparison for MoCMSCs60 and CuMoCMSCs60.

A similar phenomenon was observed at other operating scan rates of 2, 5, and 10 V s^−1^ (Figure 9b,c). As a result, CuMoCMSCs60 obtained a higher capacitance with respect to MoCMSCs60 at all operating super-high scan rates (Figure 9d). This is owing to the binary metal oxides of CuMoO_x_ equipped with multiple valence states of the copper ion and molybdenum ion. Moreover, CuMoCMSCs60 owns the merits of 2.5D binder-free electrode architecture design, the micro-/nanostructure particle synthesis, and oxygen vacancy introduction. These merits are conducive for them to obtain a high capacitance, especially at super-high operating scan rates. Therefore, CuMoCMSCs60 obtained a high capacitance of 15.4 mF cm^−2^ at 1 V s^−1^ and maintained 5.33 mF cm^−2^ at 10 V s^−1^. This scan rate of 10 V s^−1^ operated with CuMoCMSCs60 is about 50–100 times higher than that of the CuMoO_4_–graphene-composite-based supercapacitor synthesized by the hydrothermal method [77], CuMnO_2_-reduced-graphene-oxide-nanocomposite-based supercapacitor prepared by a combination of the modified Hummers method and hydrothermal strategy [78], Cu_3_Mo_2_O_9_//La_2_Mo_3_O_12_ supercapacitors fabricated by combining the co-precipitation route and annealing method [79], and the flower-shape 3D NiMoO_x_ nanomaterial for the supercapacitor prepared by a combination of the hydrothermal strategy and thermal-treatment process in an inert-gas atmosphere [48]. GCD testing was further performed to verify the electrochemical performance of MoCMSCs60 and CuMoCMSCs60. Figure 10a proved that both MoCMSCs60 and CuMoCMSCs60 achieved a symmetric GCD profile, which indicated good capacitive behavior was gained. Moreover, with respect to MoCMSCs60, CuMoCMSCs60 showed a much longer discharging time at 2 mA cm^−2^, which means a larger capacitance was obtained. Figure 10b demonstrates that the capacitance of CuMoCMSCs60 is around 4 times higher than that of MoCMSCs60 at all the operating current densities of 1.1, 1.5, 2, and 5 mA cm^−2^. This is because binary metal oxides of CuMoO_x_ facilitated a faster electronic transport rate than the single metal oxide of MoO_x_.

This superior capacitive behavior resulted from the high theoretical capacitance of CuMoO_x_, the micro-/nanostructural CuMoO_x_ particles and 2.5D electrode architectures offering plentiful active sites for ion access and improving the contact between the CuMoO_x_ active materials and electrolytes. In addition, the binder-free Mo–CuMoO_x_ integrated electrode design enriched with multiple valence states of the copper ion and molybdenum ion as well as the introduction of oxygen vacancy are conductive to improving the transport efficiency of ions and electrons. Most importantly, among all the fabricated devices, CuMoCMSCs60 equipped with the binary metal oxide microelectrode array achieved the best performance. This is attributed to the optimized machining voltage of 60 V for NCEDM facilitating the synthesis of CuMoO_x_ binary metal oxide active materials with small particles with reduced agglomerations, which provided plenty of active sites for charge storage and, thus, boosted the electrochemical performance of CuMoCMSCs60. Furthermore, this NCEDM technique facilitates the one-step fabrication of CuMoCMSCs with a tailorable capacitance without the utilization of binders, expensive conducting fillers (like graphene and carbon nanotube), harmful chemicals and solutions, additional current collectors (such as graphene-coated nickel foam and gold substrate), and inert gases (such as Ar and N_2_). In addition, the synthesized processes of binary metal oxides and fabricated procedures of MSCs were greatly simplified by the one-step NCEDM technique, which does not require the help of other material processing techniques such as stirring, dispersing, sonicating, coating, washing, filtering, and heating. This one-step and numerically controlled fabrication technique developed in this research opens a new vision for preparing BMO materials, BMO microelectrodes, and BMO microdevices in an environmental, automatic, and intelligent way.

## 4. Conclusions

To summarize, a novel method for synthesizing BMO materials and fabricating BMO microelectrodes and BMO microdevices was well-developed in this work using the NCEDM technique. This simple process resulted in the one-step fabrication of CMSCs with a customized capacitance tailored by the machining voltage and machining tool of NCEDM. The fabricated CuMoCMSCs60 with binary metal oxides could operate at an ultra-high scanning rate of 10 V s^−1^, and gained a capacity of 40.3 mF cm^−2^ (at 1.1 mA cm^−2^), which is more than 4 times higher than that of MoCMSCs60, which is owing to the synergistic effect of copper ions and molybdenum ions. Moreover, the CuMoCMSCs60 showed a better performance than CuMoCMSCs80 and CuMoCMSCs100, attributed to the low machining voltage facilitate synthesis of CuMoO_x_ with reduced agglomeration. Moreover, this superior capacitive behavior resulting from the high theoretical capacitance of CuMoO_x_, the micro-/nanostructural CuMoO_x_ particles, the 2.5D binder-free Mo–CuMoO_x_-integrated electrode design, and the introduction of oxygen vacancy are conductive to improving the transport efficiency of ions and electrons. Most importantly, this NCEDM technique facilitates the one-step fabrication of CuMoCMSCs without the utilization of binders, expensive conducting fillers, harmful chemicals and solutions, additional current collectors, inert gases, and additional synthesized processes. Therefore, it opens a new vision for preparing BMO materials, BMO microelectrodes, and BMO microdevices in an environmental, automatic, and intelligent way.

## Data Availability

The original contributions presented in the study are included in the article/Supplementary Materials; further inquiries can be directed to the corresponding authors.

## Supplementary Materials

The following supporting information can be downloaded at: https://www.mdpi.com/article/10.3390/mi15111319/s1. Figure S1. (a) XRD pattern, (b,c) XPS profiles of (b) Mo 3d3/2 and Mo 3d5/2, and (c) O 1s for Mo-MoOx integrated electrodes. Figure S2. SEM image of Mo-MoOx integrated electrodes. Table S1. Compared areal capacitances for different kinds of supercapacitors fabricated by various manufacture techniques.
