# Supplementary material for: One-Step Fabrication of 2.5D CuMoOx Interdigital Microelectrodes Using Numerically Controlled Electric Discharge Machining for Coplanar Micro-Supercapacitors
Source: Micromachines (Basel). 2024 Oct 29;15(11):1319. doi: 10.3390/mi15111319 (PMC11596134; doi:10.3390/mi15111319)
Supplement: Supplementary file 1 [file micromachines-15-01319-s001.zip › micromachines-3273549-supplementary.pdf]

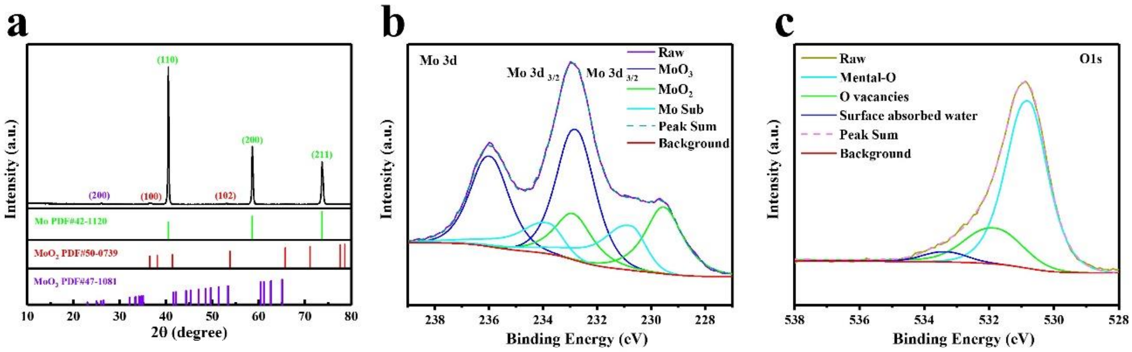

Figure S1. (a) XRD pattern, (b-c) XPS profiles of (b) Mo $3d_{3/2}$ and Mo $3d_{5/2}$, and (c) O 1s for Mo-MoO_x integrated electrodes.

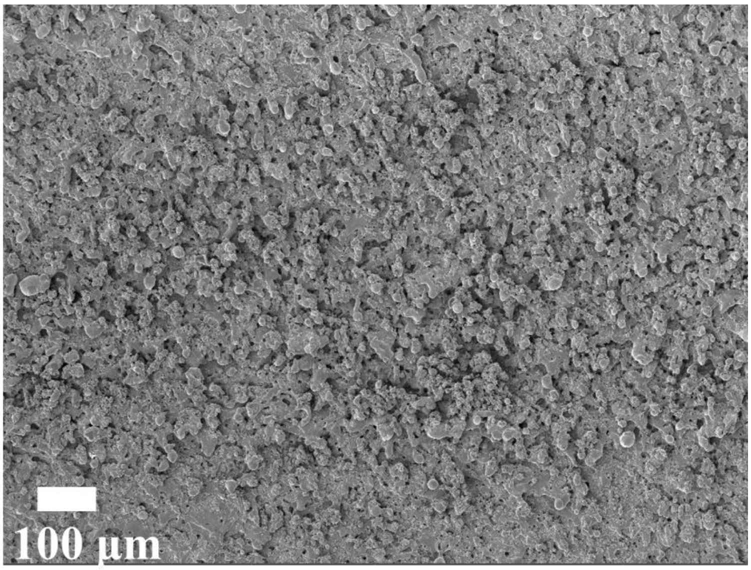

Figure S2. SEM image of Mo-MoO_x integrated electrodes.

Table S1 Compared areal capacitances for different kinds of supercapacitors fabricated by various manufacture techniques

Devices	Fabricated Techniques	Current collectors	Capacitance (mF cm^{-2})	References
$NiWO_4$// AC	Wet chemical route, Slurry impregnation	Al foil	17.01	[1]
V_2O_5-rGO MSC	Spray coating, Sputtering	Au/Cr	24	[2]
MnO_2//$NiCo_2O_4$ MSCs	Electrodeposition, Electron beam evaporation, Spin-coating	Gold	5.36	[3]
MXene MSCs	Laser machining Spray coating	Integrated electrodes	23	[4]
$CuCo_2O_4$-CNT MSCs	Hydrothermal, CO_2 laser scribing	ITO	10.88	[5]
Activated carbon MSCs	Inkjet printing, Photolithography, Chemical vapour deposition	Gold	5.1	[6]
MnO_2-carbon MSCs	Screen printing, Hydrothermal	Silver	7.04	[7]
Graphene MSCs	Spin coating, Lithography	Gold	0.08	[8]
HfO_2-graphene MSCs	Laser engraving, Sputtered deposition	Graphene	6.4	[9]
rGO MSCs	Laser radiation, Vacuum filtration	Carbon coated aluminium foils	0.51	[10]
CuMo CMSCs60	NCEDM	Integrated electrodes	41.9	**This work**

References

1. Jha, S.; Mehta, S.; Chen, Y.; Renner, P.; Sankar, S. S.; Parkinson, D.; Kundu, S.; Liang, H., NiWO 4 nanoparticle decorated lignin as electrodes for asymmetric flexible supercapacitors. *Journal of Materials Chemistry C.* **2020,** *8*, 3418-3430.
2. Boruah, B. D.; Nandi, S.; Misra, A., Layered assembly of reduced graphene oxide and vanadium oxide heterostructure supercapacitor electrodes with larger surface area for efficient energy-storage performance. *ACS Applied Energy Materials.* **2018,** *1*, 1567-1574.
3. Patil, S. J.; Park, J. S.; Kim, Y. B.; Lee, D. W., A Quasi 2D Flexible Micro-Supercapacitor Based on MnO2//NiCo2O4 as a Miniaturized Energy-Storage Device. *Energy Technology.* **2018,** *6*, 1380-1391.
4. Jiang, Q.; Wu, C.; Wang, Z.; Wang, A. C.; He, J.-H.; Wang, Z. L.; Alshareef, H. N., MXene electrochemical microsupercapacitor integrated with triboelectric nanogenerator as a wearable self-charging power unit. *Nano Energy.* **2018,** *45*, 266-272.
5. Basu, A.; Bhardwaj, M.; Gawli, Y.; Rode, C.; Ogale, S., A Robust Highly Flexible All-solid-state Micro Pseudocapacitor Based on Ternary Oxide CuCo2O4having Ultrathin Porous Nanowall Type Morphology Blended with CNT. *ChemistrySelect.* **2016,** *1*, 5159-5164.
6. Pech, D.; Brunet, M.; Taberna, P.-L.; Simon, P.; Fabre, N.; Mesnilgrente, F.; Conédéra, V.; Durou, H., Elaboration of a microstructured inkjet-printed carbon electrochemical capacitor. *Journal of Power Sources.* **2010,** *195*, 1266-1269.
7. Wang, Y.; Shi, Y.; Zhao, C. X.; Wong, J. I.; Sun, X. W.; Yang, H. Y., Printed all-solid flexible microsupercapacitors: towards the general route for high energy storage devices. *Nanotechnology.* **2014,** *25*, 094010.
8. Wu, Z. S.; Parvez, K.; Feng, X.; Müllen, K., Graphene-based in-plane micro-supercapacitors with high power and energy densities. *Nature communications.* **2013,** *4*, 2487.
9. Sain, S.; Chowdhury, S.; Maity, S.; Maity, G.; Roy, S. S., Sputtered thin film deposited laser induced graphene based novel micro-supercapacitor device for energy storage application. *Scientific Reports.* **2024,** *14*, 16289.
10. Gao, W.; Singh, N.; Song, L.; Liu, Z.; Reddy, A. L. M.; Ci, L.; Vajtai, R.; Zhang, Q.; Wei, B.; Ajayan, P. M., Direct laser writing of micro-supercapacitors on hydrated graphite oxide films. *Nature nanotechnology.* **2011,** *6*, 496-500.
